# Direct-Current Electrical Field Stimulation of Patient-Derived Colorectal Cancer Cells

**DOI:** 10.3390/biology12071032

**Published:** 2023-07-22

**Authors:** Falko Lange, Katrin Porath, Tina Sellmann, Anne Einsle, Robert Jaster, Michael Linnebacher, Rüdiger Köhling, Timo Kirschstein

**Affiliations:** 1 Oscar-Langendorff-Institute of Physiology, Rostock University Medical Center, 18057 Rostock, Germany; 2 Division of Gastroenterology and Endocrinology, Department of Medicine II, Rostock University Medical Center, 18057 Rostock, Germany; 3 Molecular Oncology and Immunotherapy, Clinic of General Surgery, Rostock University Medical Center, 18057 Rostock, Germany

**Keywords:** galvanotaxis, direct-current electrical field, colorectal cancer, patient-derived low-passage cell lines, calcium influx, PI3K/AKT pathway, Raf/MEK/ERK pathway

## Abstract

**Simple Summary:**

In colorectal carcinoma, migration of the cancer cells greatly contributes to the progression of the disease. One of the driving factors for a directional migration could be the presence of direct-current electrical fields, which is known as galvanotaxis. To investigate migration of colorectal cancer cells in direct-current electrical fields, we employed five low-passage cell lines that were derived from surgical specimens. In three out of five cell lines, a preferred cathodal migration was determined. Exposure to electrical fields in vitro had no effect on cellular integrity. Furthermore, we found voltage-gated calcium channels crucial in galvanotaxis. Intracellular signaling pathways based on the kinases MEK and AKT were identified as being involved in the migratory phenotype. We conclude that colorectal cancer cells are capable of galvanotactic migration. The directional migration was dependent on calcium influx and activation of central signaling pathways of colorectal cancer.

**Abstract:**

Several cues for a directional migration of colorectal cancer cells were identified as being crucial in tumor progression. However, galvanotaxis, the directional migration in direct-current electrical fields, has not been investigated so far. Therefore, we asked whether direct-current electrical fields could be used to mobilize colorectal cancer cells along field vectors. For this purpose, five patient-derived low-passage cell lines were exposed to field strengths of 150–250 V/m in vitro, and migration along the field vectors was investigated. To further study the role of voltage-gated calcium channels on galvanotaxis and intracellular signaling pathways that are associated with migration of colorectal cancer cells, the cultures were exposed to selective inhibitors. In three out of five colorectal cancer cell lines, we found a preferred cathodal migration. The cellular integrity of the cells was not impaired by exposure of the cells to the selected field strengths. Galvanotaxis was sensitive to inhibition of voltage-gated calcium channels. Furthermore, signaling pathways such as AKT and MEK, but not STAT3, were also found to contribute to galvanotaxis in our in vitro model system. Overall, we identify electrical fields as an important contributor to the directional migration of colorectal cancer cells.

## 1. Introduction

Colorectal carcinoma (CRC) represents the third-most-common tumor disease and is the fourth-leading cause of cancer-related deaths in the world [1,2]. In most cases, metastases are the main reason for the high rate of mortality. Common sites of metastasis are the liver and the peritoneum [3]. Metastasization is a complex multi-step process of epithelial–mesenchymal transition, migration, and invasion [4]. Migration of the cancer cells is believed to be multifactorial, driven by several stimuli, such as chemotactic agents and haptotactic and durotactic cues [5]. Another driving force for a directional migration could be galvanotaxis. Galvanotaxis (also referred to as electrotaxis) is the migration of cells along direct-current electrical fields (DCEF), experimentally presented as a permanent stimulus or in a pulsed manner [6]. Under physiological conditions, DCEF arise from transepithelial potentials (TEP) that emerge from asymmetric ion flux over plasma membranes [7,8]. As a result, the membrane of cells is depolarized on the cathodal side and hyperpolarized on the anodal one within the electrical field. Our current understanding also includes a second basic mechanism, a phenomenon termed electromigration [9,10]. It has been shown that membrane-bound electrically charged components such as receptors and ion channels may be electrophoretically distributed to the anode or cathode under DCEF conditions. Hence, via electromigration, membrane-bound receptors may predominantly cluster within the membrane on one or the other side of the cells.

Electrical fields may occur in the colonic epithelium due to its major role in chloride secretion and absorption (reviewed by Negussie et al., Cells 2022 [11]). Since net chloride secretion through basolateral NKCC1 and apical CFTR dominates transepithelial electrolyte transport in the colon mucosa [12], there is a typical lumen-negative TEP of roughly −10 mV in mice [13] and −30 mV in healthy humans [14]. Although there might be some decrease in the lumen-negative TEP along the colonic longitudinal axis, since chloride absorption processes are more relevant in the distal colon [15], it is well conceivable that the lumen-negative TEP over a single cell layer (approx. 100 µm) could create a permanent electrical field of >250 V/m. For this reason, we suppose that it could be possible for cathodic migration of colorectal cancer cells, i.e., towards the lumen, to take place in the human colon.

To the best of our knowledge, no data for the migratory behavior of CRC cells in DCEF have been published so far. However, in various solid malignancies such as brain cancer [16,17,18,19], lung cancer [20,21,22,23], prostate cancer [24], and breast cancer [25,26] migration in the DCEF has been reported. In the migration of cells, the influx of Ca^2+^ and the intracellular Ca^2+^ concentration play major roles [5], which also applies to CRC cells [27]. Interestingly, the expression of Ca^2+^-permeable ion channels has been found to be altered in CRC [28,29,30] and has been associated with tumor progression [27,31]. A high dependency on Ca^2+^ influx contributes to invasion and migration [27], but may also affect the directional motility in electrical fields [32].

Therefore, we hypothesize that CRC cells exhibit a directional migration in the DCEF. As a biological CRC in vitro model, we employed a panel of patient-derived low-passage cell lines that were established from primary tumors and a metastasis from surgical CRC patients. In the current study, the cells were challenged with different electrical field strengths, and their migration in 2D galvanotaxis chambers was determined. Moreover, the role of Ca^2+^ influx and the impact of signaling pathways such as PI3K/AKT, Raf/MEK/ERK, and STAT3 on directional migration in the electrical field were investigated.

## 2. Materials and Methods

### 2.1. Patient-Derived Low-Passage Cell Lines

Primary CRC resection specimens and a liver metastasis were obtained from surgery at the clinic of general surgery of the Rostock University Medical Center, with informed written patient consent. All procedures were approved by the Ethics Committee of the Rostock University Medical Center (reference numbers II HV 43/2004 and A 45/2007) in accordance with generally accepted guidelines for the use of human material. Establishment of the cell line HROC18 [33] and the other four cell lines has been described in detail before [34]. The in vitro models were either directly established from freshly taken tumor material (HROC18, HROC173, HROC383) or following xenografting (HROC277, HROC277Met2) in immunodeficient mice [35]. All primary tumors were obtained from untreated adenocarcinoma (Table 1). Additionally, HROC277Met2, a cell line derived from a liver metastasis obtained from subsequent surgery of the patient with tumor ID HROC277 was employed in the study. Of the selected CRC models, all but HROC383 were of a microsatellite stable subtype (Table 1). More detailed information on the HROC models can be found in Mullins et al. 2019 [35].

To culture HROC cell lines, Dulbecco’s Modified Eagle Medium: Nutrient Mixture F-12 (DMEM/F12; from PAN Biotech, Aidenbach, Germany) with 10% fetal calf serum (FCS, Bio&SELL, Feucht, Germany) was used. Culturing of the cells was done at 37 °C in a 5% CO_2_ humidified atmosphere. All experiments were performed with ≤50 cell passages. At constant intervals, MycoSPY^®^—PCR Mycoplasma Test Kit (Biontex, Munich, Germany) was used to test cell culture supernatants for mycoplasma contamination. No contamination with mycoplasmas were found during the project period.

### 2.2. Quantification of DNA Synthesis

To gauge the effects of small-molecule inhibitors and calcium channel blockers on proliferation of the cells, a 5-bromo-2′-deoxyuridine (BrdU) incorporation assay kit (Roche Diagnostics GmbH, Mannheim, Germany) was performed as surrogate. In our study, MEK inhibitor trametinib, AKT inhibitors capivasertib and MK-2206, and STAT3 inhibitor niclosamide (all from Selleck Chemicals, Houston, TX, USA) were employed. DMSO was used as a solvent. Additionally, the effects of MgCl_2_, NiCl_2_, and verapamil (Tocris bioscience, Bristol, UK; all dissolved in DMEM/F12) were investigated. The cells (5 × 10^3^/well) were seeded in 96-well half-area microplates at equal densities and allowed to adhere overnight in a complete culture medium. On the following day, the culture medium was replaced by a medium supplemented with inhibitors, MgCl_2_ or NiCl_2_ at the indicated doses. After an incubation period of 32 h, BrdU was added at a final concentration of 10 µM into the culture media. Another 16 h later, labeling was stopped, and BrdU uptake was measured according to the manufacturer’s instructions.

### 2.3. Migration in the Direct-Current Electrical Field

To determine the galvanotactic migration of CRC cells, cultures were exposed to direct-current electrical fields (DCEF) as described [19]. For this purpose, the HROC cells (2 × 10^4^) were seeded onto collagen I-coated (Advanced Biomatrix, San Diego, CA, USA) coverslips that were mounted into direct-current (DC) chambers. After 24 h, silver/silver chloride electrodes were placed into the outer reservoirs. Current flow was conducted by agar bridges consisting of 2% agarose (TopVision agarose, ThermoScientific, Waltham, MA, USA) in Ringer’s solution (Braun, Melsungen, Germany). A DC power supply (Standard Power PackP25, Biometra, Göttingen, Germany) was used to apply current for 6 h. During this time, voltage was determined directly at the borders of the cell area, with a 25 mm distance in-between, with a multimeter (Voltcraft VC220, Conrad Electronic, Wollerau, Switzerland) and adjusted during the experiments to maintain a constant electrical field strength.

The positions of the cells prior to and after 6 h of DC stimulation were determined by analyses of microphotographs at eight fixed positions distributed over the whole area, which were taken at the beginning and end of the experiment, employing a Leica DMI 6000 microscope (Leica, Wetzlar, Germany) with Leica Application Suite (v. 2.0.0.13332) software package. An exact overlay of both microphotographs, taken at the start and end, was brought about using the image software GIMP (2.10.30). Subsequently, these pictures were exported for evaluation of cell migration in ImageJ (1.53e).

In ImageJ, five to six cells per field of view were analyzed by encircling cells, including all cell extensions and centering cells. In total, 40 cells per chamber were analyzed. Circle center coordinates were determined at the start (X_0_/Y_0_) and six hours later (X_1_/Y_1_). Based on these coordinates, the distance of each dimension in the two-dimensional system and overall migration distance ($d=\sqrt{{\left(X_{0}-X_{1}\right)}^{2}+{\left(Y_{0}-Y_{1}\right)}^{2}}$) were calculated (this value is referred to as the absolute value of migration; see single-cell data of 0 V/m and 200 V/m in Supplementary Figure S1). The DC electrical field was oriented along the X-axis, with the positive X-axis towards the anodal and the negative X-axis towards the cathodal pole. All n-numbers given in the text and figures correspond to the number of cells that were analyzed in three or more biological independent experiments. To test different strengths of DCEF conditions, three field strengths (150 ± 15 V/m, 200 ± 20 V/m, 250 ± 25 V/m) were applied, all of which ranged within previously used field strengths of in vitro studies [16,18,19,36,37,38].

To examine the impact of Ca^2+^ influx on galvanotaxis, HROC cell cultures were challenged with MgCl_2_, NiCl_2_ or verapamil for the entire duration of the experiment. MgCl_2_ was used to block Ca^2+^ influx in an unspecific manner. To inhibit Ca^2+^ flux via L-Type Ca^2+^ channels, verapamil was applied. To impair T-type Ca^2+^ channels, the cell cultures were exposed to NiCl_2_. Effects of signal transduction pathways associated with migration of CRC cells were addressed employing MK-2206, capivasertib, trametinib or niclosamide. The selected doses of MK-2206 [39,40], capivasertib [41], trametinib [42,43], and niclosamide [44,45] had previously shown antitumoral effects in various in vitro cancer models.

### 2.4. Fluorescence Microscopy

To estimate cellular integrity after DC stimulation, nuclei and cytoplasm were stained. Therefore, HROC cells were cultured on coverslips as described above. After DC stimulation for 6 h, the coverslips were washed with phosphate-buffered saline (PBS) and fixed afterwards in 3.7% paraformaldehyde overnight at 4 °C. The next day, slides were washed again in PBS and permeabilized with 0.5% TritonX-100 (Carl Roth, Karlsruhe, Germany). After another washing step with PBS, cellular actin was stained using Acti-stain 488 phalloidin (Cytoskeleton, Denver, CO, USA; diluted 1:140 in PBS) for 30 min at room temperature. Subsequently, the cells were washed and embedded with ProLong^TM^ Gold containing DAPI (ThermoFisher Scientific, Waltham, MA, USA) on microscope slides. Fluorescence analysis was performed by laser-scanning microscopy (Leica DMI 6000) with Leica Application Suite (v. 2.0.0.13332) software.

### 2.5. Statistical Analysis

Statistical analysis was performed with SigmaPlot 13.0. The experimental results are presented in box plots or given as mean ± standard error of the mean (SEM) for the indicated number of cells and experiments. Mean group differences were tested for significance using the nonparametric Kruskal–Wallis test before, for multiple comparisons, subgroups were tested with a post hoc Dunn’s test. To analyze migration velocity and field strength, we used a two-way ANOVA followed by a Bonferroni *t*-test. A significance level of *p* < 0.05 was considered to be statistically significant.

## 3. Results

### 3.1. Migration of CRC Cells in the DC Eletcrical Field

**Figure 1:**
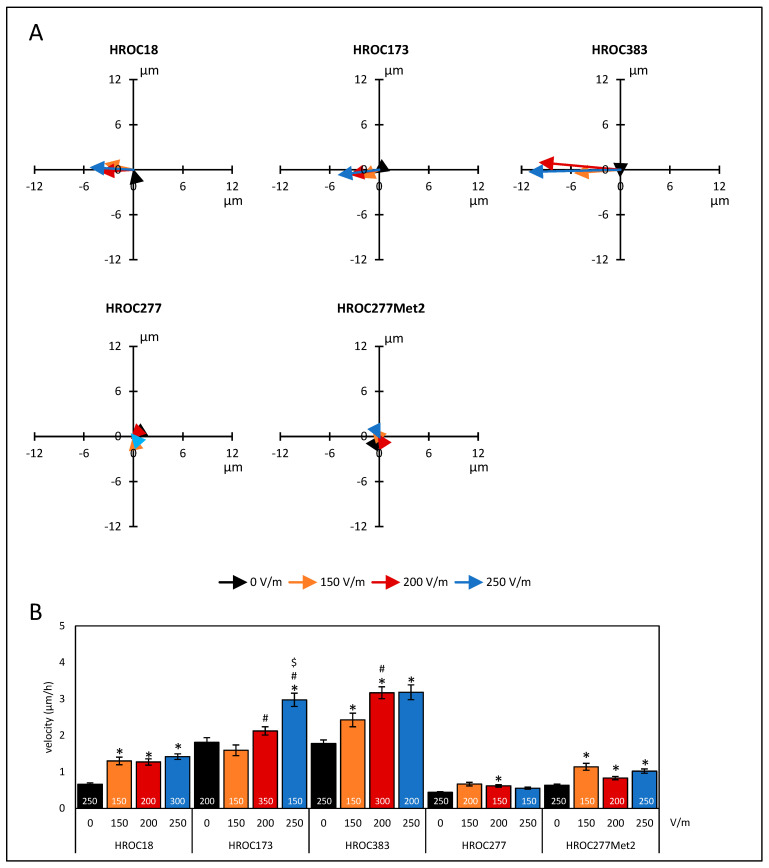
Migration of colorectal cancer cells under DC electrical field conditions. CRC cells (HROC18, HROC173, HROC383, HROC277, and HROC277Met2) were seeded on collagen-coated coverslips that were mounted in DC chambers. The exact positions of the cells prior to and after DC stimulation were estimated by analyses of microphotographs at eight fixed positions. At each field of view, positions in the two-dimensional system of five to six cells were determined. The distances along the field vectors in the X-dimension and the Y-dimension (perpendicular to the field vectors) and overall migration velocity were calculated. (**A**) Sum vectors of total distance after six hours of DC stimulation with 150 V/m (orange), 200 V/m (red), 250 V/m (blue), and control cultures *w*/*o* DC (black). Positive values in the X-dimension represent anodal migration; negative vales imply a cathodal movement of the cells. (**B**) Velocities of tumor cells under different DC conditions. Data are represented as mean velocity ± SEM; 150–350 cells for each experimental group were analyzed (exact numbers are given in the columns) based on 3–7 independent experiments; * *p* < 0.05 versus 0 V/m; # *p* < 0.05 versus 150 V/m; $* p* < 0.05 versus 200 V/m (Kruskal–Wallis test with post hoc Dunn’s test).

Five patient-derived colorectal cancer cell lines were employed in our study. In initial studies, the cells were challenged with three different field strengths to test migratory behavior in the direct-current (DC) electrical field (DCEF). Sum vectors showed a motile phenotype independently of DC stimulation (Figure 1A). After DC stimulation based on a field strength of 150 V/m, HROC18 (0 V/m: 0.66 ± 0.04 µm/h vs. 150 V/m: 1.31 ± 0.11 µm/h) and HROC277Met2 (0 V/m: 0.63 ± 0.04 µm/h vs. 150 V/m: 1.14 ± 0.09 µm/h) presented enhanced velocities (*p* < 0.05 for all, Kruskal–Wallis test with post hoc Dunn’s test; Figure 1B). In both cell lines, a further increase of field strengths had no effect on the migratory velocity of the tumor cells. In HROC173, a field strength of 200 V/m was required to increase velocity in comparison to unstimulated controls (0 V/m: 1.81 ± 0.13 µm/h vs. 200 V/m: 2.12 ± 0.11 µm/h). However, significant differences in the range of 150–250 V/m were found (Figure 1B). Cell line HROC383 presented a stepwise increase of velocity ranging from 0–200 V/m (0 V/m: 1.78 ± 0.1 µm/h vs. 150 V/m: 2.42 ± 0.19 µm/h, 0 V/m vs. 200 V/m: 3.17 ± 0.17 µm/h; 150 V/m vs. 200 V/m). A further increase of the DCEF to 250 V/m had no effect on migration velocity. In contrast, in HROC277, only at a field strength of 200 V/m, a small but significant increase in velocity was found (0 V/m: 0.44 ± 0.01 µm/h vs. 200 V/m: 0.62 ± 0.04 µm/h).

Additionally, a two-way ANOVA (factor tumor origin, i.e., HROC277 vs. HROC277Met2, and factor current, ranging from 0–250 V/m) with Bonferroni post hoc test revealed that the metastasis cell line HROC277Met2 exhibited a significantly higher migration velocity, regardless of the selected field strength, than did the cell line HROC277 derived from the primary tumor (*p* < 0.001; two-way ANOVA followed by Bonferroni *t*-test).

**Figure 2:**
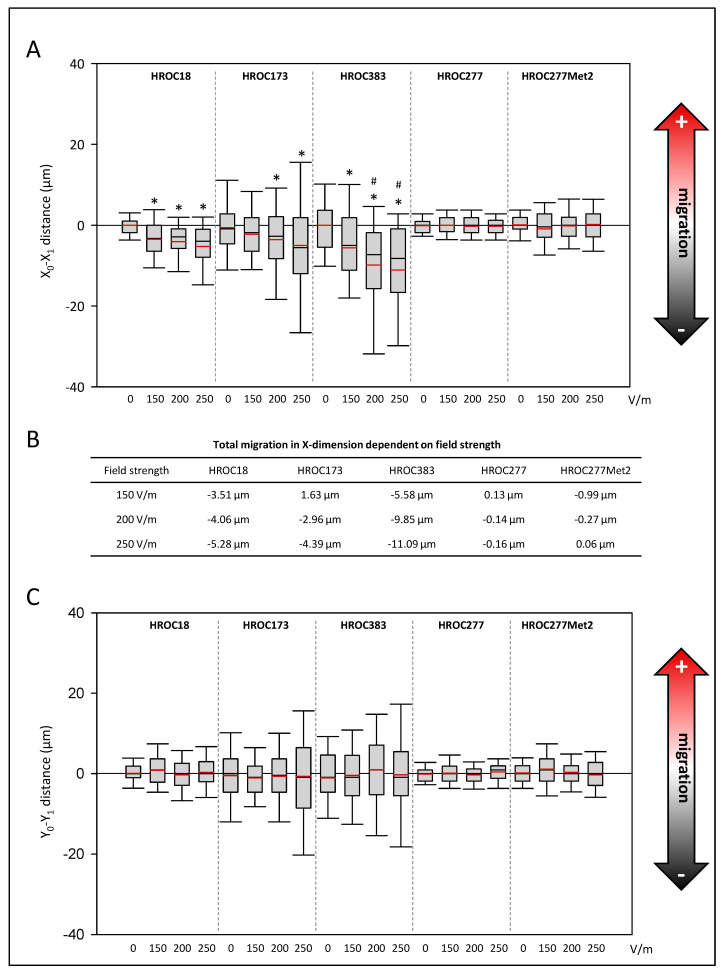
Effects of DC electrical fields on pole-directed migration of CRC cells. CRC cells (HROC18, HROC173, HROC383, HROC277, and HROC277Met2) were stimulated with DC fields ranging from 0–250 V/m for six hours, and pole-directed migration ((**A**) X-dimension, (**C**) Y-dimension) was calculated based on analyses of microphotographs at eight fixed positions on each slide. For each experimental group, 150–350 cells were analyzed based on 3–7 independent experiments. Median is shown as a black-colored line and the mean is red. (**B**) Total migration in X-dimension was calculated as difference values of DC stimulated cell cultures minus values of control cultures; * *p* < 0.05 versus control cultures *w*/*o* DC; # *p* < 0.05 versus 150 V/m DC (Kruskal–Wallis test with post hoc Dunn’s test).

Regarding the migration along vectors of the DCEF, we examined whether the cells migrated preferentially to the anodal or cathodal pole. For this purpose, the distance of migration in the X-dimension from the beginning to the end of the experiment was calculated (Figure 2A). Cell lines HROC18, HROC173, and HROC383 significantly migrated in the cathodal direction (*p* < 0.05, Kruskal–Wallis test with post hoc Dunn’s test). Furthermore, in HROC383, an enhanced migration towards the cathode between 150 V/m-stimulated cultures and those exposed to higher field strengths was found (*p* < 0.05, Kruskal–Wallis test with post hoc Dunn’s test). With respect to the absolute values (difference of DC stimulation minus control conditions), the HROC383 cell line presented the overall highest distance (11.09 µm) of migration at 250 V/m (Figure 2B). Neither HROC277 nor the metastasis cell line derived from the same patient presented a galvanotactic phenotype.

With respect to the Y-dimension, no significant differences were found among all the selected cell lines (Figure 2C).

**Figure 3:**
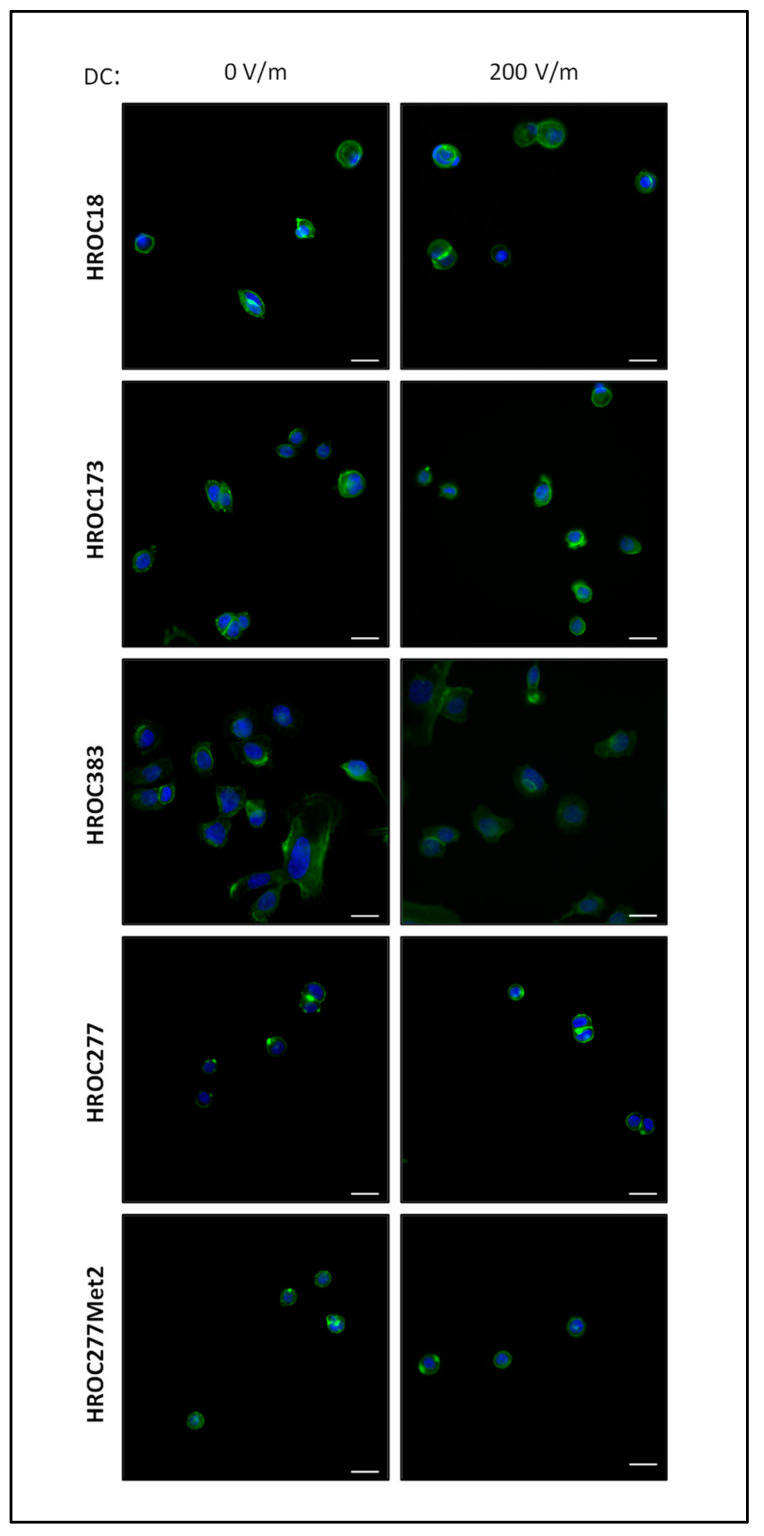
Fluorescence microscopic images of HROC cell cultures after DC stimulation. HROC cells were exposed to a field strength of 200 V/m for 6 h, or were cultured under control conditions *w*/*o* DC stimulation for the same amount of time. Afterwards, Actin was visualized using Acti-stain 488 phalloidin (diluted 1:140 in PBS; shown in green). Nuclei were counterstained with DAPI (blue). Representative images are based on microscopic photographs that were taken at 400× magnification. Bars represent 25 µm.

Next, we investigated whether DCEF conditions might affect cellular integrity and viability of our in vitro models, as apoptosis was reported in previous studies (reviewed in [46]). As illustrated by microscopic analysis of cell cultures stimulated with field strength of 200 V/m in comparison to control cultures, no impairments such as apoptotic bodies or nuclear fragmentation were identified (Figure 3; see Supplementary Figures S2–S6 for high-resolution microscopic photographs). Also, there was no abundance of stress fibers in the CRC cells.

### 3.2. Ca^2+^ Influx Is Mandatory for Directional Migration of HROC383 Cell Cultures in the DCEF

**Figure 4:**
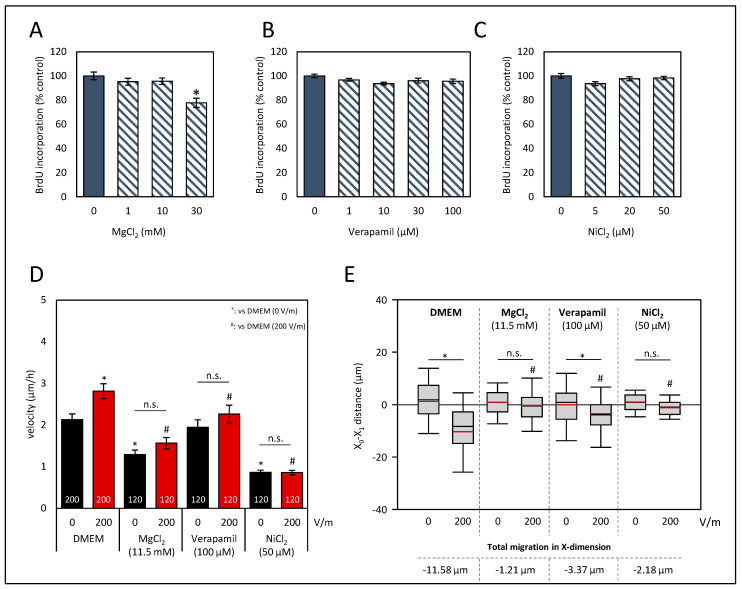
Effects of Ca^2+^ influx perturbation on proliferation and galvanotaxis of HROC383 cell cultures. (**A**–**C**) HROC383 cells growing in 96-well half-area microplates were exposed to MgCl_2_, verapamil or NiCl_2_ at the indicated doses for 48 h before DNA synthesis was assessed with the BrdU incorporation assay. One hundred percent BrdU incorporation corresponds to cells cultured with solvent (cell culture medium) only. Data are presented as mean ± SEM (n ≥ 12 separate cultures); * *p* < 0.05 versus control cultures (Kruskal–Wallis test with post hoc Dunn’s test). (**D**) The velocity of tumor cells under DCEF conditions (200 V/m, 6 h) and control conditions. Cells were treated with MgCl_2_, verapamil, NiCl_2,_ and DMEM, respectively, at the indicated doses. Data are represented as mean velocity ± SEM; 120–200 cells for each experimental group from three to five independent experiments were analyzed; n.s., no significant difference; * *p* < 0.05 vs. DMEM *w*/*o* DC; # *p* < 0.05 vs. DMEM with 200 V/m DC (Kruskal–Wallis test with post hoc Dunn’s test). (**E**) The pole-directed migration was estimated, and total migration in X-dimension was calculated as the difference in the values of DC-stimulated cell cultures and the values of control cultures. n.s., no significant difference; * *p* < 0.05 vs. control cultures *w*/*o* DC; # *p* < 0.05 vs. DMEM with 200 V/m DC (Kruskal–Wallis test with post hoc Dunn’s test).

Since we aimed to contribute to a better understanding of molecular mechanisms of galvanotaxis in CRC, we tested whether Ca^2+^ influx is mandatory. To that end, the HROC383 cell line with the highest response to the DCEF was selected as a viable in vitro model. In our experiments, we focused on an intermediate field strength of 200 V/m, as no further increase was achieved with a higher stimulation (Figure 2A). In prior experiments based on DCEF stimulation, BrdU assays were performed to exclude potential harmful doses (Figure 4). Three different conditions to impair transmembraneous Ca^2+^ influx into the HROC383 cells were used in our study. MgCl_2_ was employed as an unspecific blocker of transmembraneous Ca^2+^ influx. To impair ion flux via T-type Ca^2+^ channels, NiCl_2_ was used. A third condition included the L-Type Ca^2+^-channel blocker verapamil. At the selected doses in experiments regarding galvanotaxis (11.5 mM MgCl_2_, 100 µM verapamil, and 50 µM NiCl_2_), no effects on cell proliferation were determined (Figure 4A–C).

Our data show that both MgCl_2_ and NiCl_2_ not only prevented a DC-stimulated increase of the velocity of HROG383 cells (Figure 4D), but also reduced the velocity in comparison to solvent-treated cultures (*p* < 0.05, Kruskal–Wallis test with post hoc Dunn’s test). The latter finding was not surprising, given that calcium influx is mandatory for cellular migration [47]. Interestingly, the addition of verapamil had no effect on the velocity of cultures *w*/*o* DC stimulation (Figure 4D). However, no increase in the velocity after DC stimulation was determined and, in comparison to DC-stimulated control cultures, velocity was also found to be reduced (*p* < 0.05, Kruskal–Wallis test with post hoc Dunn’s test).

As illustrated in Figure 4E, X-dimensional migration was analyzed. Both treatments, that with MgCl_2_ and that with NiCl_2_, abolished galvanotaxis of HROC383 cells (*p* < 0.05, Kruskal–Wallis test with post hoc Dunn’s test). Exposure of the cells to verapamil did not entirely prevent cathodal movement, but in comparison to control cultures *w*/*o* verapamil, X-dimensional migration was significantly reduced (*p* < 0.05, Kruskal–Wallis test with post hoc Dunn’s test). With respect to the Y-dimension, no significant differences were found (Supplementary Figure S7). In summary, our data indicate that Ca^2+^ influx greatly contributes to a directional migration in galvanotaxis.

### 3.3. Effects of Intracellular Signaling Cascades on Galvanotaxis of HROC383 Cell Cultures

In addition to Ca^2+^ flux over the plasma membrane, various intracellular signaling pathways are known to contribute to galvanotaxis. Based on studies on other tumor entities [17,19,48,49] and keratinocytes [50], we proposed that the phosphatidylinositol 3-kinase (PI3K)/AKT pathway could be a key player in the directional migration of HROC383 cells in DCEFs. Therefore, two small-molecule kinase inhibitors that act as specific AKT blockers were chosen for investigation (allosteric AKT inhibitor MK-2206 and capivasertib, an ATP-competitive inhibitor). Furthermore, the closely linked Raf/MEK/ERK pathways may also contribute to galvanotaxis [48]. To specifically target this pathway, trametinib, a MEK inhibitor, was selected. A third pathway in CRC that has been associated with migration is based on signal transducer and activator of transcription 3 (STAT3) [51], which could be targeted with niclosamide [52].

**Figure 5:**
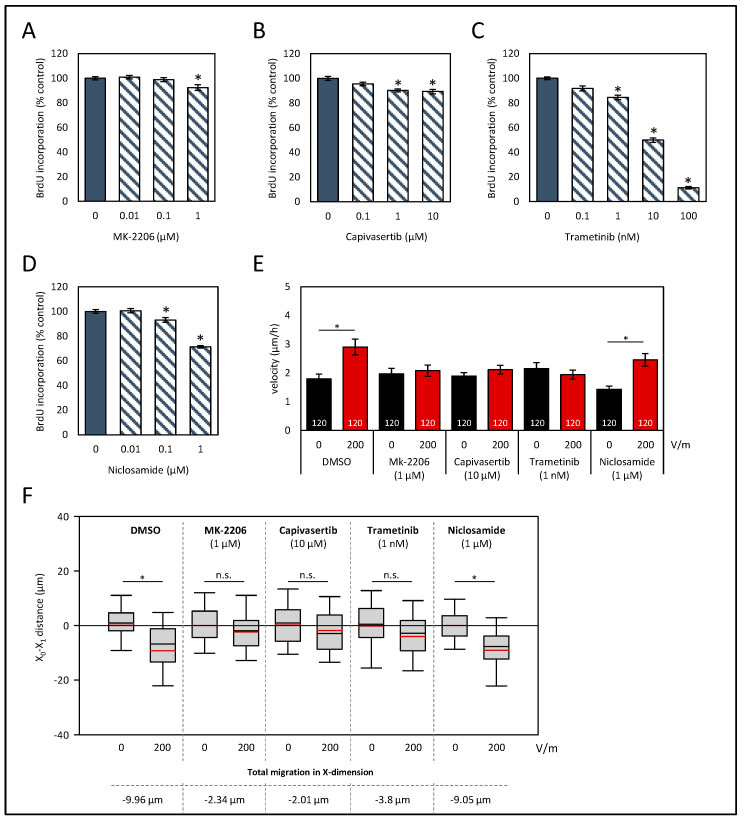
Effects of signaling pathway inhibitors on proliferation and galvanotaxis of HROC383 cells. (**A**–**D**) HROC383 cells growing in 96-well half-area microplates were treated with AKT inhibitors MK-2206 or capivasertib, MEK inhibitor trametinib, or STAT3 inhibitor niclosamide at the indicated doses for 48 h, before DNA synthesis was assessed with the BrdU incorporation assay. One hundred percent BrdU incorporation corresponds to cells cultured with solvent only. Data are presented as mean ± SEM (n ≥ 12 separate cultures); * *p* < 0.05 versus control cultures (Kruskal–Wallis test with post hoc Dunn’s test). (**E**) The velocities of tumor cells under DCEF conditions (200 V/m, 6 h) and control conditions. Cells were treated with inhibitors or solvent (DMSO), respectively, at the indicated doses. Data are represented as mean velocity ± SEM. As illustrated in the columns, 120 cells for each experimental group from three independent experiments were analyzed; * *p* < 0.05 versus control *w*/*o* DC (Kruskal–Wallis test with post hoc Dunn’s test). (**F**) The pole-directed migration was estimated, and total X-dimension migration was calculated. * *p* < 0.05 vs. control cultures *w*/*o* DC; n.s., no significant difference; (Kruskal–Wallis test with post hoc Dunn’s test).

Again, assays on proliferation were performed to determine well-tolerated doses that presented only minor biological effects (Figure 5A–D). Based on these studies, 1 µM MK-2206, 10 µM capivasertib, 1 nM trametinib, and 0.1 µM niclosamide were selected for subsequent analysis.

In solvent-treated cell cultures, DC stimulation of 200 V/m led to an increase in the velocity of the cells (Figure 5E; *p* < 0.05, Kruskal–Wallis test with post hoc Dunn’s test). All cell cultures, except for those exposed to niclosamide, lacked an increase in velocity after DC stimulation. Regarding the pole-directed migration, both AKT inhibitors and trametinib abolished the cathodal migration (Figure 5F). Exposure of the cells to the STAT3 inhibitor niclosamide had no effect on galvanotaxis. Here, as in in DC-stimulated control cultures (−9.96 µm), a high cathodal migration of roughly −9 µm was observed. No significant differences regarding a Y-dimensional migration were determined (Supplementary Figure S7B).

## 4. Discussion

To an increasing extent, the impact of electrical fields as a major driving force in migration of cells has been recognized. With respect to CRC, previous studies on migration have focused on environmental cues based on chemical or physical signals, while, to the best of our knowledge, investigation on the motility of CRC cells in DC electrical fields has been neglected so far.

The results of this study showed that CRC cells exhibit a galvanotactic phenotype. Based on patient-derived in vitro models that were established from primary tumors, three out of four in vitro models presented a cathodal migration under DCEF conditions. In principle, in the DCEF, cells may migrate in a directional manner (cathodal or anodal pole) or show no directness at all. In the DCEF, the minus pole attracts cations outside of the cells, and the transmembrane potential decreases. As a result, the membrane becomes further depolarized on these parts of the cells, whereas on the anodal side, the membrane becomes hyperpolarized due to an asymmetric ion influx via voltage-gated ion channels [36]. So far, it has not yet been conclusively clarified which biophysical mechanisms account for the predominant direction [53,54]. Of the five selected patient-derived cell lines, only HROC383 was classified as MSI-high, whereas all other cell lines were of a MSS subtype. Since the cells of both subtypes showed a cathodal migration, no implication of a link between the molecular status and galvanotaxis was suggested.

Our data indicate an important role for voltage-gated Ca^2+^-permeable ion channels in CRC cells, since these greatly contributed to the directional migration in HROC383 cell cultures. The unspecific Ca^2+^ influx was abolished by high levels of MgCl_2_ in the culture media, whereas Ca^2+^ influx via T-Type Ca^2+^ channels was inhibited by NiCl_2_ substitution. Furthermore, in experiments, including ones involving the L-Type Ca^2+^ channel blocker verapamil, cathodal migration of HROC383 was found only to be attenuated and not entirely blocked. Nonetheless, galvanotaxis was found to be reduced in comparison to the untreated control. As Ca^2+^-permeable channels are often found to be upregulated in CRC (summarized in Ref. [55]), which results in high intracellular Ca^2+^ levels [56], influx via these ion channels could play an important role in galvanotaxis of this tumor entity. The mechanism of Ca^2+^ influx as a response to DC stimulation is complex and far from full understanding [53,54]. In human osteoblasts, inhibition of T-type and L-type Ca^2+^ channels had no effect on galvanotaxis, but store-operated channels (SOCs) were identified as crucial [57]. In contrast, Babona-Pilipos and colleagues reported that inhibition of T-type and L-type Ca^2+^ channels reduced the velocity of neural precursor cells but did not affect the directness of the migration [58]. Another study, on murine fibroblasts, described an attenuation of a cathodal migration by inhibition of SOCs and T-type Ca^2+^ channels, but not for L-type channels [59]. Furthermore, Ca^2+^ influx via Ca^2+^ carriers (e.g., Na^+^/Ca^2+^ exchangers, Ca^2+^ ATPases), and the potential role of Ca^2+^-permeable TRP channels have been neglected so far. In addition, hyperpolarization of the plasma membrane on the anodal side may attract intracellular Ca^2+^ by passive electrochemical diffusion. As a result, the cytoplasmic Ca^2+^ concentration on the anodal side of the cell may increase, and thus may substantially contribute to the ion distribution in the cell [6]. Furthermore, in a small set of experiments, an electrophoretic distribution of membrane-bound receptors and ion channels within the DCEF was reported, which may additionally alter the migratory behavior [20,60]. Therefore, further studies that systematically investigate the role of Ca^2+^-mediated galvanotaxis need to be conducted to elucidate the molecular mechanisms, an effort which may also help to identify future targets for the treatment of CRC.

In our study, we were able to include two cell models that were established from the same patient: HROC277, based on tissue of the primary tumor, and HROC277Met2, which was derived from a liver metastasis one year later. Interestingly, both cell lines presented no galvanotaxis at all. However, we found an increase in the motility of the HROC277Met2 cell cultures. As both cell lines were exposed to the same field strengths, one may speculate that the redistribution of ion channels and altered opening properties may orchestrate an overall higher velocity of the metastasis-derived cells. The overall low response to DCEFs is in line with the principal author’s previously published data on the migration of a brain metastases cell line derived from CRC [19].

Other findings included that a DCEF of 200 V/m seems to have had no immediate effect on the cellular integrity of the HROC cultures, and that no hint on direct induction of nuclear fragmentation as part of the apoptosis cascade was found. The field strengths of 150–250 V/m (transferred to the cellular level ~200 µV/µm) are in a physiological range that can be assumed to be present in the colon in vivo. Apoptosis induction seems highly dependent on the cell type and experimental conditions (e.g., field strength and environment) [54]. Field strengths up to 400 V/m in smooth muscle cells of rabbits [61], human keratinocytes [62], and olfactory bulb neural progenitor cells [63] did not lead to apoptosis, or even prevented cell death. In contrast, in the B16 melanoma cell line, induction of apoptosis by application of high-voltage (7.5 kV/m) pulses was reported [64]. Moreover, in other studies based on high-voltage electrical fields, apoptosis was frequently detected [65,66,67].

Furthermore, we asked whether signaling pathways that were associated with the motility of CRC cells and galvanotaxis in various tumor entities may also affect the directional migration in our patient-derived in vitro model. The PI3K/AKT pathway was identified as a key player in galvanotaxis in various cell entities [17,19,48,49,68]. Based on experiments using two AKT inhibitors with different mechanisms of action, we could also validate the comprehensive role of this pathway in galvanotaxis for CRC. In agreement with previously published studies on brain cancer cells [48,49], keratinocytes [69], and corneal epithelial cells [9], perturbation of the Raf/MEK/ERK pathway results in an impairment of DC-stimulated migration. Interestingly, Huang et al. 2016 reported only an effect on the motility of the cancer cells, but, after inhibition of MEK1/2, not in the directionality [49]. Of the five selected patient-derived cell lines, only HROC383 cells harbored a mutation in B-Raf [35]. Oncogenic B-Raf was reported to contribute to an increased migration of CRC cells [70,71]; therefore, the mutation in HROC383 may also be a reason for the large, directed migration of this CRC model. In line with reported studies [52,72,73,74], niclosamide exhibited antitumoral effects on CRC cells in a dose-dependent manner. However, the migratory behavior in the DCEF was not impaired. In marked contrast, in lung cancer cells, STAT3 was identified as crucial for galvanotaxis [21]. After stimulating human T cells with a DCEF of 150 V/m, a decrease in the phosphorylation of STAT3 was determined [75]. The authors linked this finding to the polarization of the immune cells.

Since transepithelial potentials (TEP) could be the primary source of DC electrical fields in the colon, targeting key players that give rise to the asymmetric ion distribution could be of value in preventing a directed migration of CRC cells. The lumen-negative TEP is amiloride-sensitive [14]; it could be an intriguing question whether amiloride may promote intraepithelial infiltrative tumor growth. It is attempting to speculate whether an increased anion secretion and/or cation absorption could constrain intramural tumor cell infiltration.

Our study shed light on electrical fields as novel cues for the directional migration of CRC cells. The process of directed cell migration is driven by several stimuli. Hence, various signals must be integrated into the total outcome of the direction and velocity of the cells. As our study is based on in vitro 2D cultures, follow-up investigations should subsequently expand the experimental approaches to include 3D in vitro studies, or even in vivo models of CRC, in which several cues of migration may affect a migratory phenotype.

## 5. Conclusions

In summary, in patient-derived CRC cell lines, we demonstrated for the first time that these cells could be stimulated to a cathodal migration in DCEFs under in vitro conditions. Our data indicate that Ca^2+^ influx in general, and specifically via voltage-gated Ca^2+^ channels, highly contributed to galvanotaxis. In addition, inhibition of the signaling pathways PI3K/AKT and Raf/MEK/ERK abolished cathodal migration, whereas signaling via STAT3 did not seem to be involved in the galvanotactic migration of CRC cells.

## Figures and Tables

**Table 1:** Colorectal cancer and metastasis cell lines [35].

Tumor ID	Gender/Age	Tumor Location	Tumor Type	Molecular Subtype ^1^
HROC18	F/65	right colon	adenocarcinoma	MSS
HROC173	M/45	left colon	adenocarcinoma	MSS
HROC277	M/77	right colon/cecum	adenocarcinoma	MSS
HROC277Met2	M/78	liver	metastasis	MSS
HROC383	F/83	colon (transverse)	adenocarcinoma	MSI-high

^1^ MSS: microsatellite stable; MSI-high: high microsatellite instability.

## Data Availability

The data presented in this study are available on reasonable request from the corresponding author.

## Supplementary Materials

The following supporting information can be downloaded at: https://www.mdpi.com/article/10.3390/biology12071032/s1, Figure S1: Galvanotaxis of CRC cells in the DE electrical field. Figure S2: Fluorescence microscopic images of HROC18 cells (original magnification ×630). Figure S3: Fluorescence microscopic images of HROC173 cells (original magnification ×630). Figure S4: Fluorescence microscopic images of HROC383 cells (original magnification ×630). Figure S5: Fluorescence microscopic images of HROC277 cells (original magnification ×630). Figure S6: Fluorescence microscopic images of HROC277Met2 cells (original magnification ×630). Figure S7: Y-dimensional migration of HROC383 colorectal cancer cells in the direct-current electrical field (DCEF).
