# Supplementary material for: Direct-Current Electrical Field Stimulation of Patient-Derived Colorectal Cancer Cells
Source: Biology (Basel). 2023 Jul 22;12(7):1032. doi: 10.3390/biology12071032 (PMC10376471; doi:10.3390/biology12071032)
Supplement: Supplementary file 1 [file biology-12-01032-s001.zip › biology-2419318-supplementary.pdf]

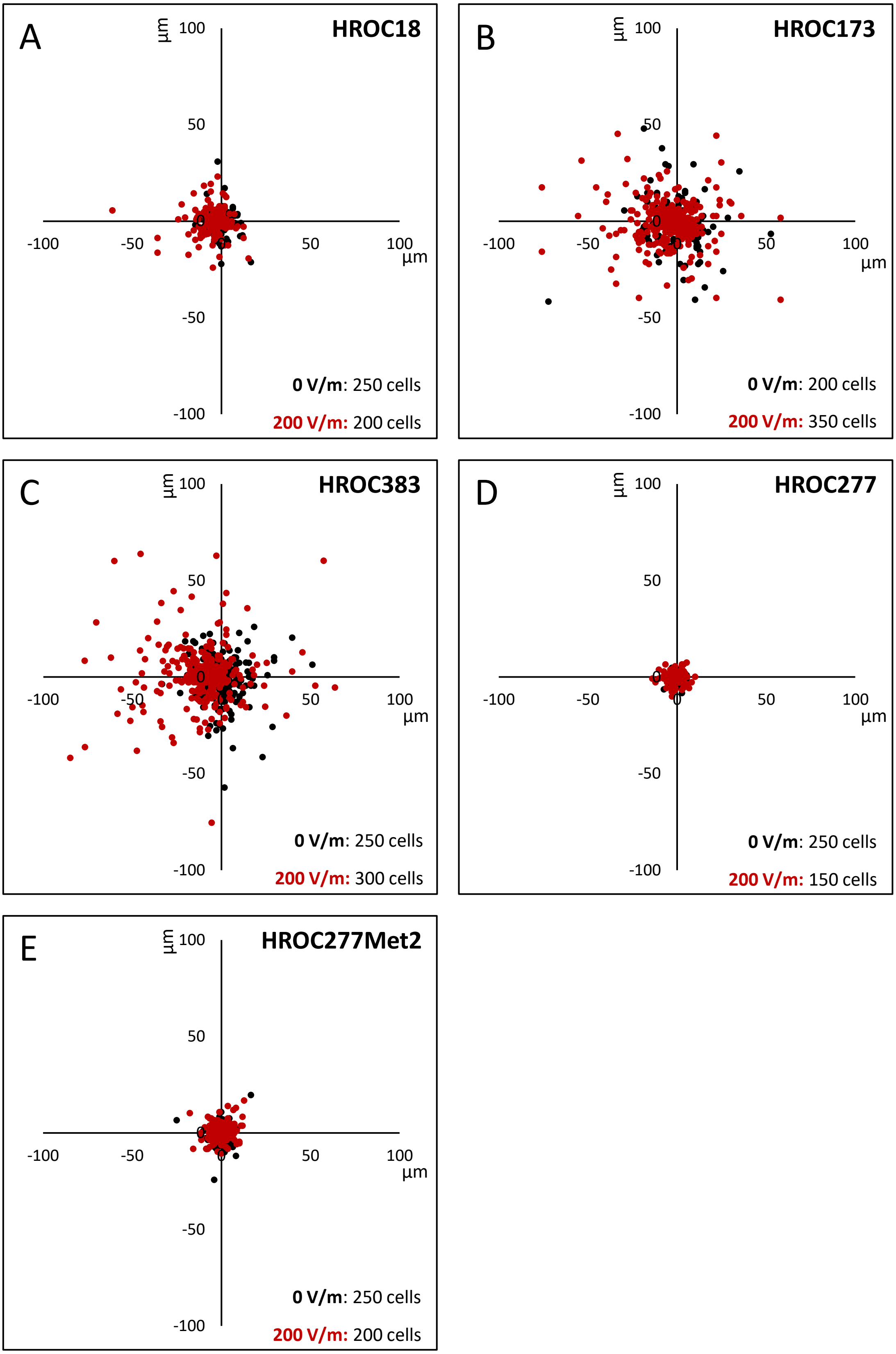

Supplementary Figure S1: Galvanotaxis of CRC cells in the DC electrical field. Migration of (**A**) HROC18, (**B**) HROC173, (**C**) HROC383, (**D**) HROC277, and (**E**) HROC277Met2 CRC cells after 6 hours of DC electrical field stimulation (200 V/m) is shown in red. Control cultures w/o DC are marked in black. In X-dimension, a migration towards positiv values indicates an anodal movement of the cells, negative values represent a cathodal migration.

HROC18

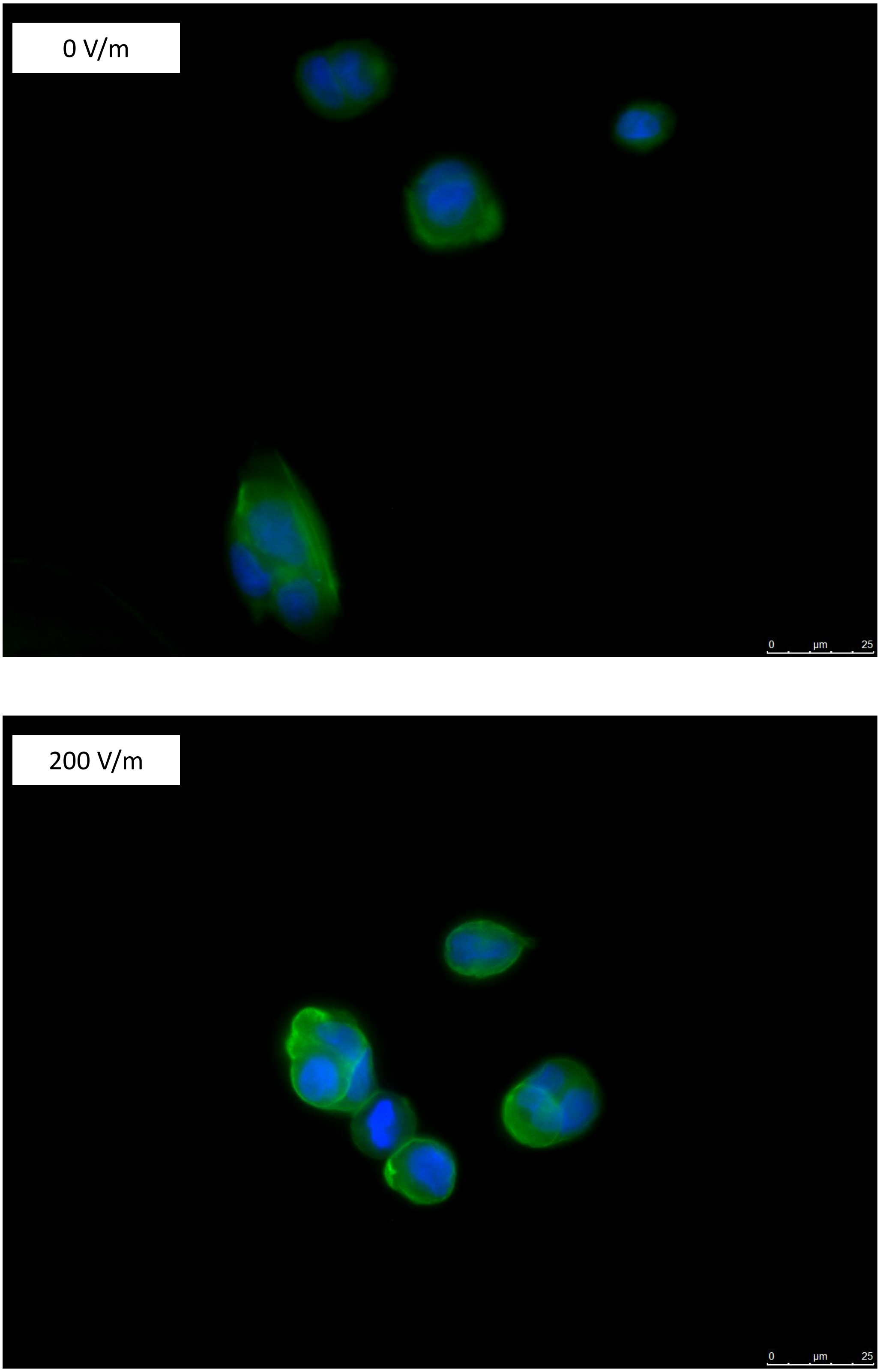

Supplementary Figure S2: Fluorescence microscopic images of HROC18 cells (original magnification ×630). Actin was visualized with Acti-stain 488 phalloidin (shown in green). Nuclei were counterstained with DAPI (shown in blue).

HROC173

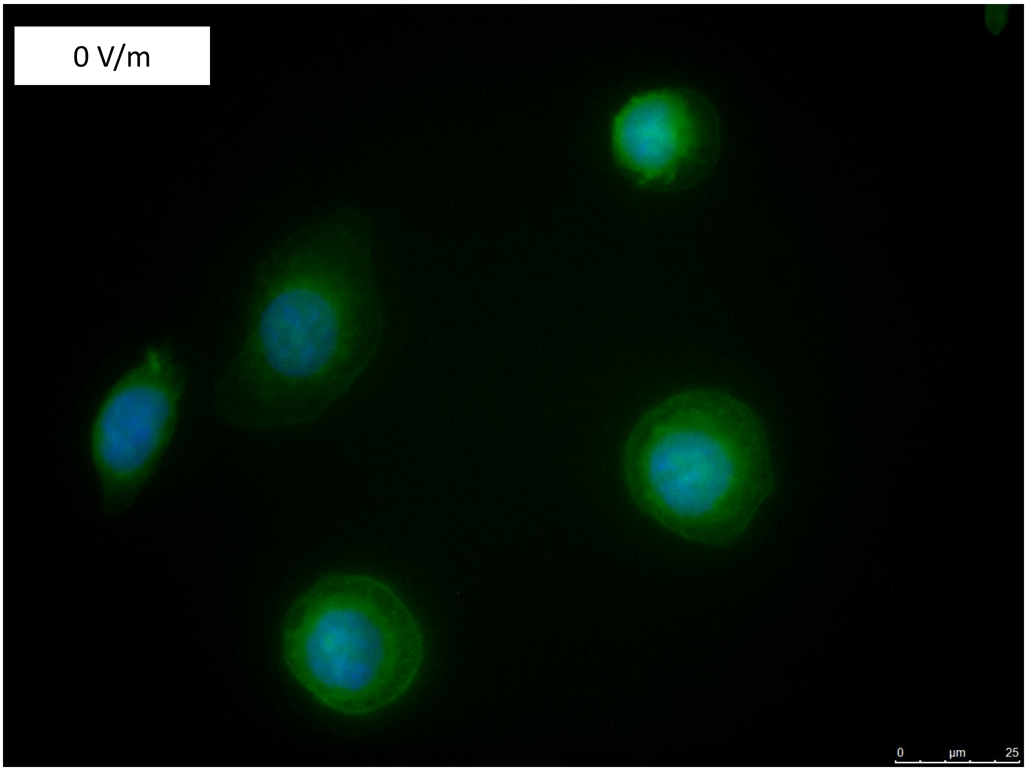

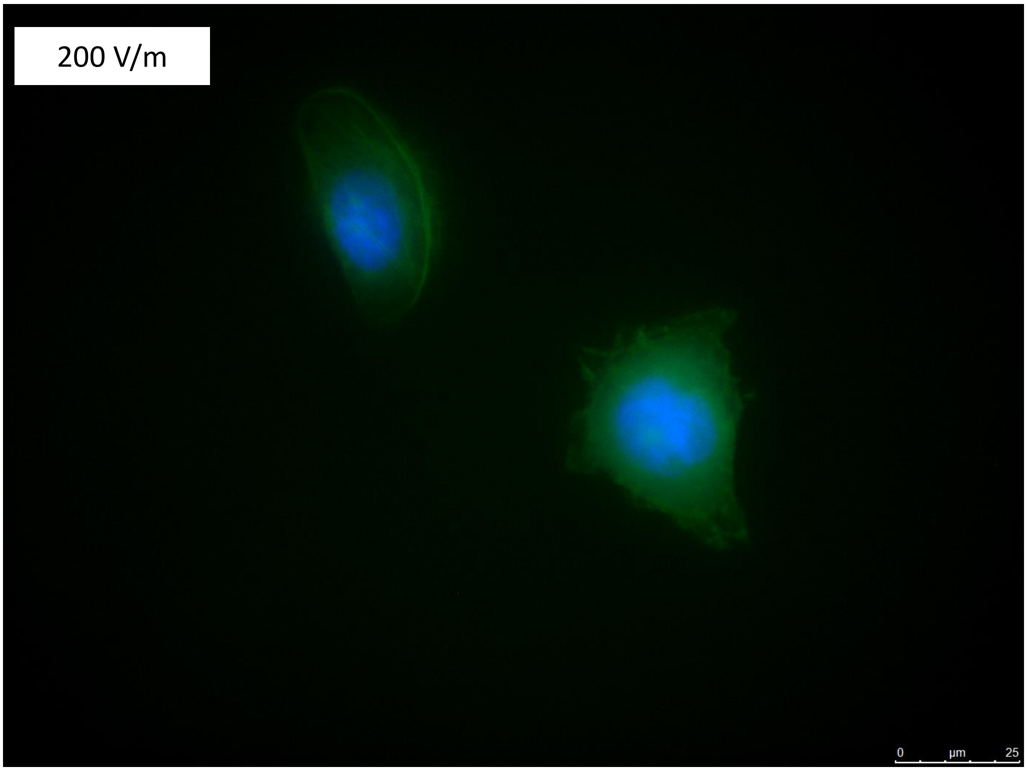

Supplementary Figure S3: Fluorescence microscopic images of HROC173 cells (original magnification ×630). Actin was visualized with Acti-stain 488 phalloidin (shown in green). Nuclei were counterstained with DAPI (shown in blue).

HROC383

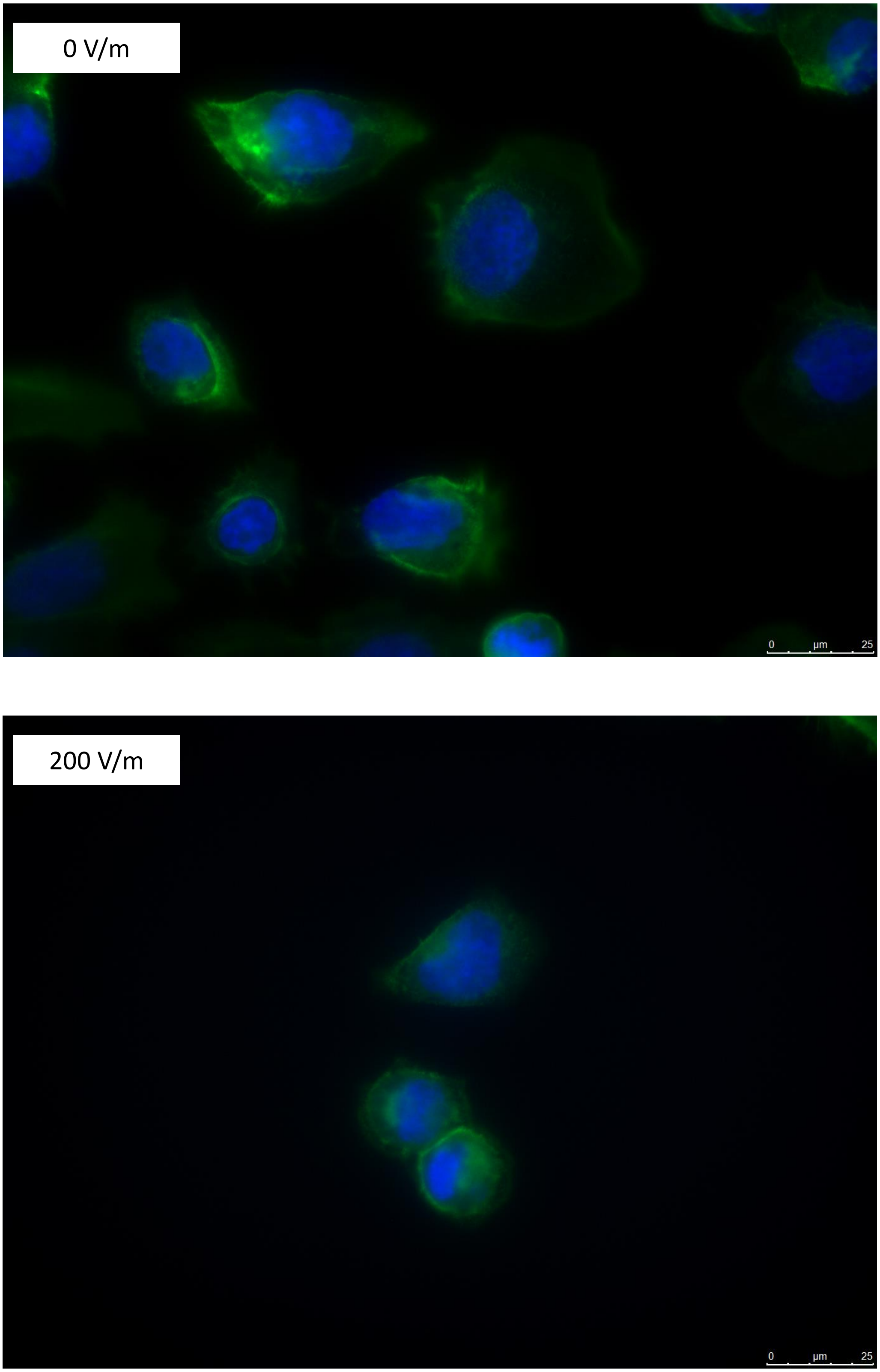

Supplementary Figure S4: Fluorescence microscopic images of HROC383 cells (original magnification ×630). Actin was visualized with Acti-stain 488 phalloidin (shown in green). Nuclei were counterstained with DAPI (shown in blue).

HROC277

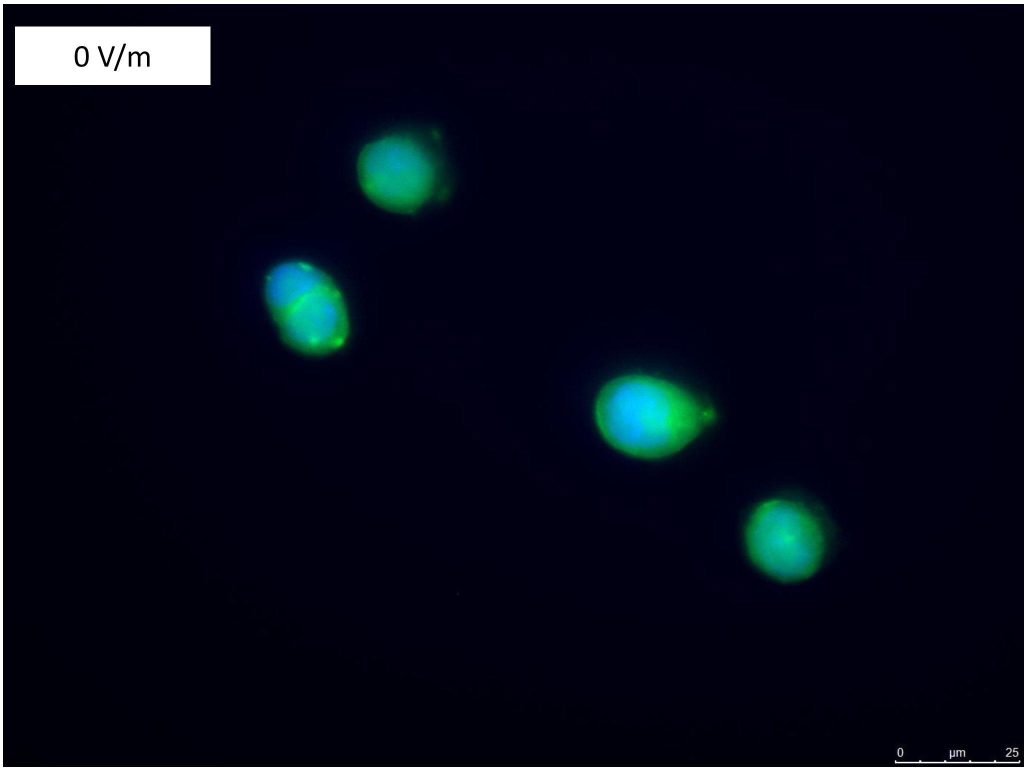

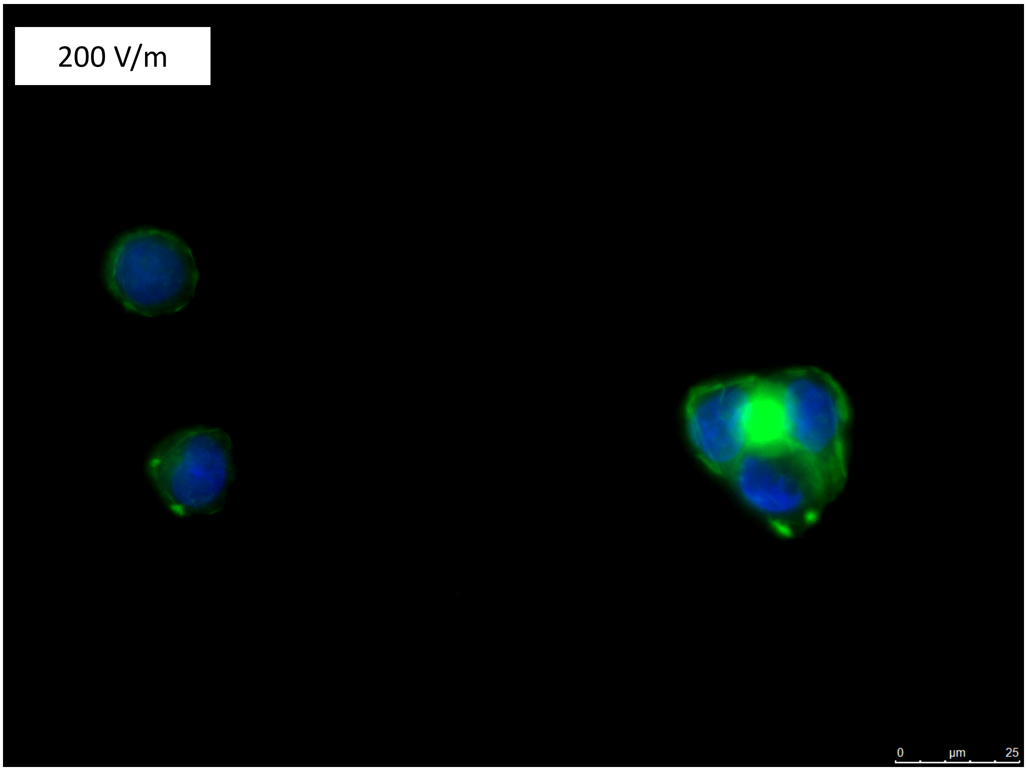

Supplementary Figure S5: Fluorescence microscopic images of HROC277 cells (original magnification ×630). Actin was visualized with Acti-stain 488 phalloidin (shown in green). Nuclei were counterstained with DAPI (shown in blue).

HROC277Met2

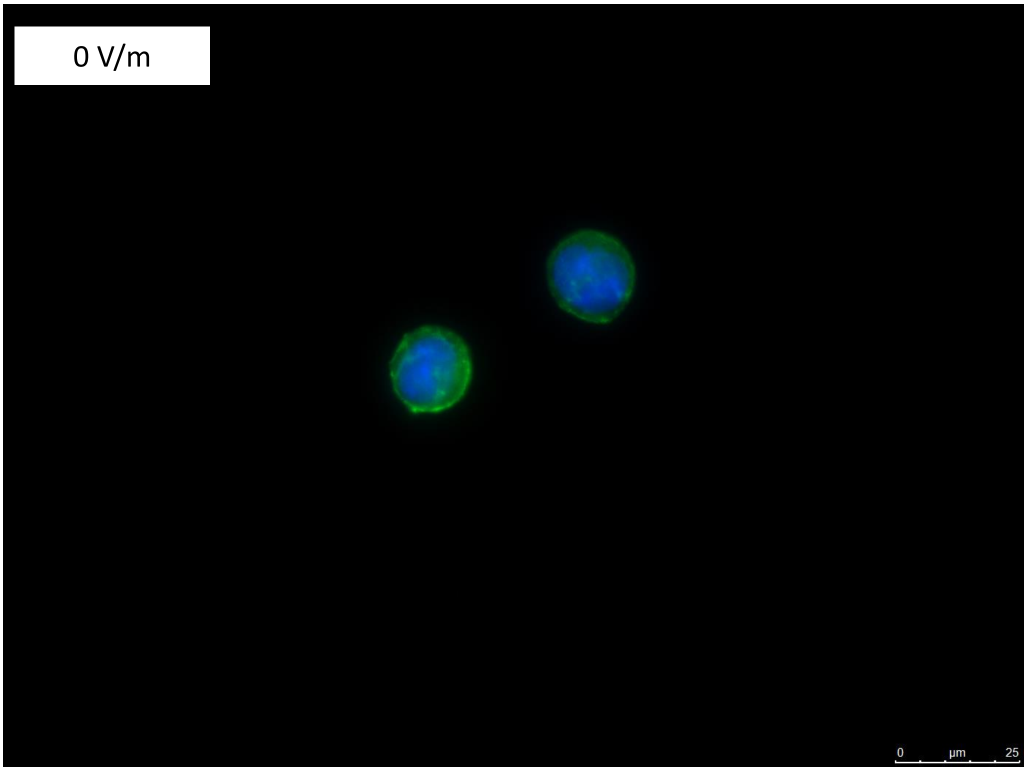

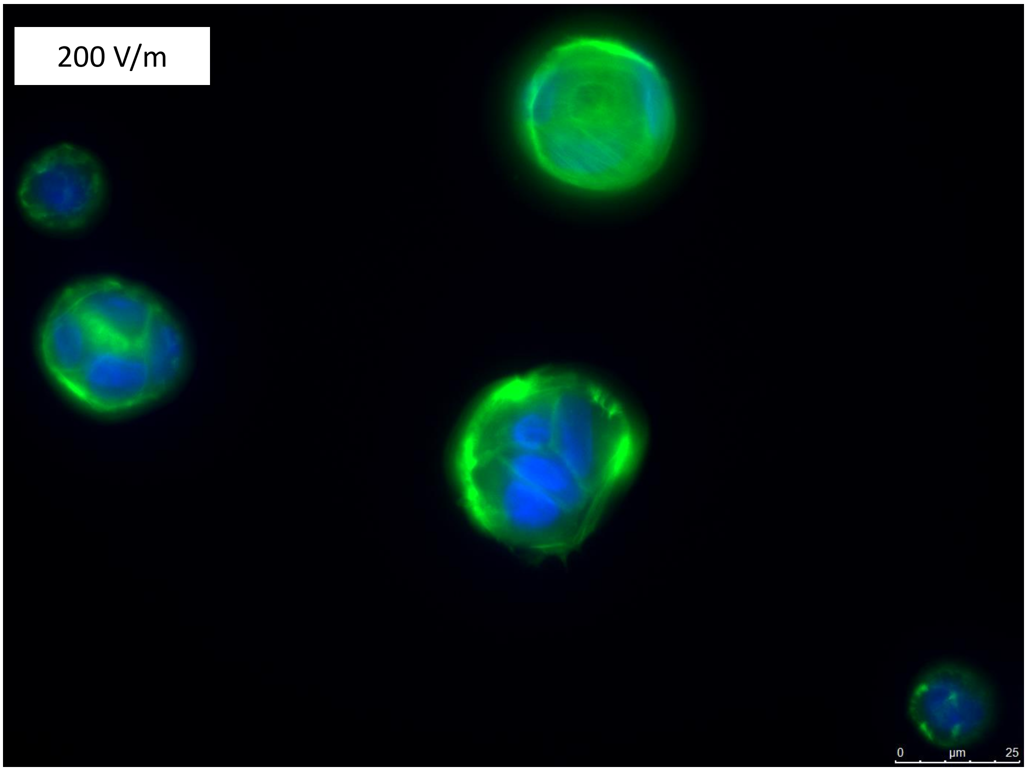

Supplementary Figure S6: Fluorescence microscopic images of HROC277Met2 cells (original magnification ×630). Actin was visualized with Acti-stain 488 phalloidin (shown in green). Nuclei were counterstained with DAPI (shown in blue).

A

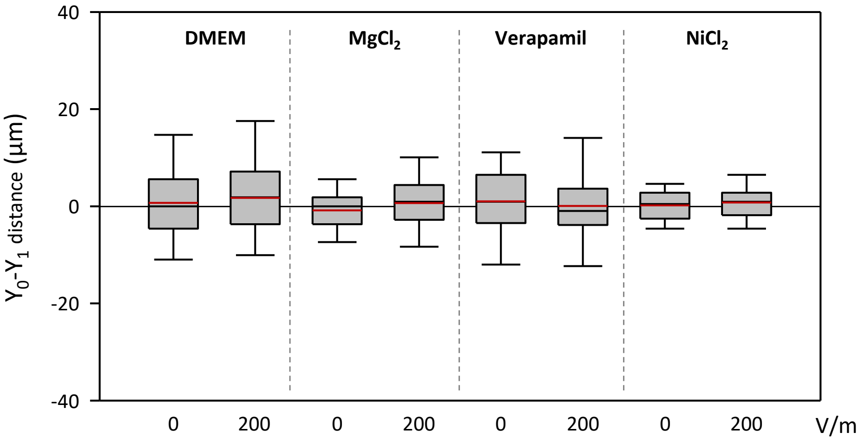

B

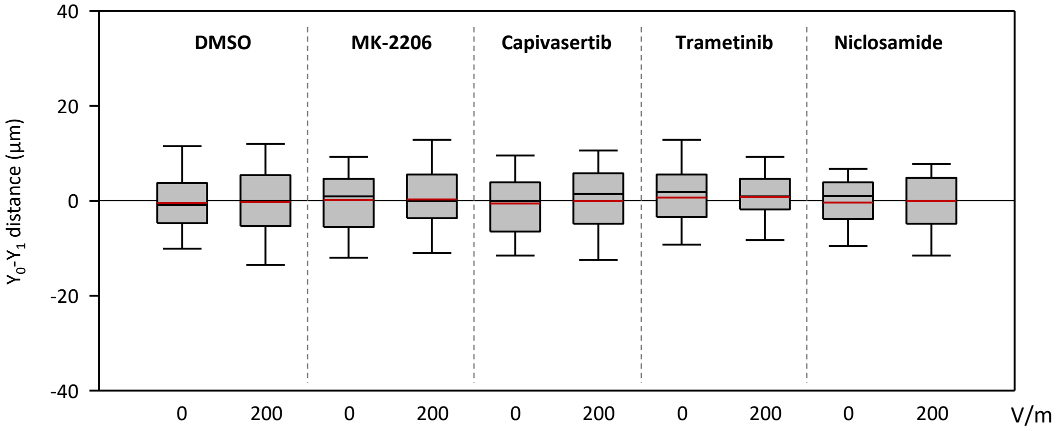

Supplementary Figure S7: Y-dimensional migration of HROC383 colorectal cancer cells in the direct-current electrical field (DCEF). Cells were stimulated for 6 hours and distance perpendicular to the DCEF (Y_0-Y_1) was determined as described in the Materials and Methods section. Cells were either exposed to **(A)** verapamil, $MgCl_2$ and $NiCl_2$ that interfere with calcium influx or **(B)** inhibitors that address intracellular signaling pathways. For each experimental group, migration of 120-200 cells was analyzed. No significant differences between control cultures w/o DC and DC-stimulated cultures were determined (Mann–Whitney U test).
